# The Role of Diet and the Gut Microbiota in Reactive Aggression and Adult ADHD—An Exploratory Analysis

**DOI:** 10.3390/nu16142174

**Published:** 2024-07-09

**Authors:** Babette Jakobi, Chiara Cimetti, Danique Mulder, Priscilla Vlaming, Barbara Franke, Martine Hoogman, Alejandro Arias-Vasquez

**Affiliations:** 1 Department of Human Genetics, Donders Institute for Brain, Cognition and Behavior, Radboud University Nijmegen Medical Center, 6525 GA Nijmegen, The Netherlands; babette.jakobi@radboudumc.nl (B.J.); chiara.cimetti2@unibo.it (C.C.); danique.mulder@radboudumc.nl (D.M.); p.vlaming@erasmusmc.nl (P.V.); barbara.franke@radboudumc.nl (B.F.); martine.hoogman@radboudumc.nl (M.H.); 2 Department of Psychiatry, Donders Institute for Brain, Cognition and Behavior, Radboud University Nijmegen Medical Center, 6525 GA Nijmegen, The Netherlands; 3 Department of Internal Medicine, Erasmus MC, University Medical Center, 3015 GD Rotterdam, The Netherlands; 4 Department of Cognitive Neuroscience, Donders Institute for Brain, Cognition and Behavior, Radboud University Nijmegen Medical Center, 6525 GA Nijmegen, The Netherlands

**Keywords:** diet, gut microbiota, ADHD, reactive aggression

## Abstract

Attention-deficit/hyperactivity disorder (ADHD) is a common neurodevelopmental condition, of-ten persistent into adulthood and accompanied by reactive aggression. Associations of diet and the gut-microbiome with ADHD as well as emotional behaviors suggest potential clinical rele-vance of both. However, studies on diet and the gut-microbiome in human reactive aggression are lacking, and should investigate the interaction between diet and the gut-microbiome leading to behavioral changes to assess their potential clinical relevance. In this study, we investigated the interaction of diet and gut-microbiota with adult ADHD and reactive aggression in 77 adults with ADHD and 76 neurotypical individuals. We studied the relationships of ADHD and reactive ag-gression with dietary patterns, bacterial community and taxonomic differences of 16S-sequenced fecal microbiome samples, and potential mediating effects of bacterial genus abundance on signifi-cant diet-behavior associations. The key findings include: (1) An association of high-energy intake with reactive aggeression scores (*p*_FDR_ = 4.01 × 10^−02^); (2) Significant associations of several genera with either reactive aggression or ADHD diagnosis with no overlap; and (3) No significant mediation effects of the selected genera on the association of reactive aggression with the high-energy diet. Our results suggest that diet and the microbiome are linked to reactive aggression and/or ADHD individually, and highlight the need to further study the way diet and the gut-microbiome inter-act.

## 1. Introduction

Attention-deficit/hyperactivity disorder (ADHD) is a common neurodevelopmental condition [1]. Symptoms of hyperactivity/impulsivity and inattention persist into adulthood in more than half of the affected individuals, and at least 15% still meet the full diagnostic criteria [2]. Up to 70% of adults with persistent ADHD symptoms are affected by emotion regulation problems, such as reactive aggression [2]. Aggressive behavior is a frequent catalyst for diagnostic consultation [3] and has a large impact on social and functional impairment, like dysfunctional relationships, peer rejection, impairments in school/occupation, and a higher risk of engaging in criminal behavior or suicidal attempts [4,5,6,7]. Little is known about the potential mechanisms underlying the co-occurrence of ADHD with reactive aggression. Alterations of the immune response, inflammatory processes affecting brain function and development [8,9], and altered neurotransmission and brain functioning in ADHD might play a role in the development of aggressive behavior. People with ADHD were shown to exhibit altered brain development in regions of emotion regulation [10,11] and altered brain functioning during emotion processing in relation to elevated reactive aggression [12]. Next to genetic predisposition, these processes are likely influenced by environmental factors [13,14,15]. Recently, diet and the gut microbiome have received attention in research on ADHD and emotional behavior, representing potential targets for prevention and treatment support [16,17,18,19]. 

Multiple studies have reported altered eating behavior in children and adolescents with ADHD compared to neurotypical peers (for a review, see [20]). While symptoms of ADHD, inattention, and impulsivity, as well as poor planning skills, may influence food choices and cause difficulties in adhering to a healthy eating pattern [17], diet might influence ADHD symptoms as well. For example, a Western diet, high in energy sources (fats, proteins, and sugars), as well as the low consumption of nutritious foods (fruits, vegetables, and foods that are rich in fiber, polyunsaturated fatty acids (PUFAs), and minerals), were associated with an increased risk for ADHD symptoms (for a review, see [17]). Furthermore, some dietary interventions could partially ameliorate the symptoms of ADHD by either restricting sugar consumption, imposing additive- and preservative-free, or hypoallergenic diets ([21] for a review see [22]), or adding supplements (e.g., omega-3 PUFAs, minerals like zinc and iron, and multivitamins [20]). Similar dietary patterns—increased consumption of sweet drinks and foods, and lower consumption of fruits and vegetables—have been associated with (emotional) self-regulation difficulties and negative emotions [23,24,25,26]. A recent study has suggested the potential protective effects of a diet rich in vegetables, fruit, and high-quality protein for aggressive behavior in men [27]. Studies on the supplementation of vitamins, minerals, and in particular omega-3 PUFAs have shown a reduction in reported incidents and aggressive behavior in imprisoned adults and in children displaying behavioral problems (for a review see [16]). Low omega-3 PUFA plasma levels in adolescents with ADHD have also been associated with atypical brain functioning during emotion processing, proposing a mechanism in which diet influences the emergence of emotion dysregulation leading to reactive aggression in ADHD [28]. However, research on the role of diet in (reactive) aggression is still underrepresented and fails to integrate the potential role of key mechanisms such as the gut microbiota [16]. 

Diet might affect reactive aggression and ADHD either via the enteric nervous system or indirectly by mediating changes in the gut microbiota [29]. The gut microbiota can influence brain functioning [30], development, and behavior relevant for ADHD and reactive aggression, e.g., by modulating the synthesis and bioavailability of key neurotransmitters such as dopamine and serotonin [31], or through neuroinflammatory processes. Food intake influences not only the growth of beneficial or pathogenic bacteria by providing their habitat and resources, but also impacts their functionality by providing the building blocks for bacterial fatty acid production and mucus production/degradation, relevant for immune system activation, or neurotransmitter or hormone synthesis, pathways associated with ADHD and aggressive behaviors [32]. Studies in children and adolescents with ADHD have reported differences in the gut microbiota composition and diversity compared to neurotypical individuals and associations of the abundance of specific bacterial genera with symptom severity (for a review, see [18]). Alterations to the gut microbiota in ADHD and their potential effects on biological pathways relevant to reactive aggression suggest shared gut microbial alterations with reactive aggression. Despite implications of the gut microbiota as a risk factor for the development of emotion dysregulation in infants [33], associations with affective disorders [34], and aggression in other species (e.g., dogs, rodents, and Drosophila [35,36,37]), there are no empirical studies in humans investigating this topic (for a review see [16]). However, Carbia and colleagues (2021) proposed a microbiome–neuro–immuno–affective framework, linking the effects of microbial alterations, inflammation, and alcohol consumption to emotional dysregulation through fronto–limbic circuits and the induction of addiction [38]. This framework supports the role of alterations to the gut microbiota, inflammation, and dietary effects in emotion dysregulation.

Despite the potential relevance of diet for reactive aggression in ADHD and the narrative overlap between dietary patterns relevant for both behaviors, to our knowledge, no study has investigated the role of diet in reactive aggression and ADHD together, or the potential effects of the gut microbiota on their relationship [16,19]. In this study, we therefore investigated the direct associations between diet and behavior (reactive aggression and ADHD) and the potential mediator role of the gut microbiota in diet–behavior relationships. We aimed to answer the following research questions: (1) Are there unique or shared dietary patterns that are associated with ADHD and reactive aggression? (2) Are there unique or shared patterns of gut microbiome diversity and composition related to ADHD and reactive aggression? (3) Do gut microbiota mediate potential diet-ADHD and/or diet-reactive aggression associations?

## 2. Materials and Methods

### 2.1. Participants and Experimental Procedure

A total of 83 adults with and 79 without ADHD participated in the IMpACT2-NL study, a Dutch cohort belonging to the International Multi-Center Persistent ADHD CollaboraTion; for a description of the study see [12]. Adult participants, older than 18 and younger than 60 years of age, were recruited from the area of Nijmegen, The Netherlands (2017–2020). Exclusion criteria were self-reported diagnoses of neurological disorders, psychosis, and/or substance abuse in the last 6 months, current major depression, and psycho-pharmaceutical therapy other than ADHD medication, confirmed with a short form from the SCID and medication questionnaires; see [12] for more detailed recruitment information. Participants were recruited for the ADHD group if they had been diagnosed with ADHD by a clinician. To confirm a diagnosis and assess the number of previous and current symptoms, we conducted the Diagnostic Interview for Adult ADHD (DIVA 2.0 [39]) in all participants. This questionnaire consists of 2 subscales of 8 inattention symptoms and 8 hyperactivity/impulsivity symptoms. Participants were included in the ADHD group if they scored ≥ 5 symptoms in one subscale and in the control group if they had no previous diagnoses of ADHD, no first-degree family members with ADHD, and <5 symptoms over both DIVA subscales [40]. All participants provided written informed consent before participating in the study. This study was approved by the local medical ethical committee (Central Commission for Human Rights Research (CCMO), NL47721.091.14, protocol 2014-290). Among a battery of neuropsychological tests and questionnaires, all participants completed a short semiquantitative food questionnaire and the Reactive-Proactive Aggression Questionnaire (RPQ) [41]. For a description of these questionnaires and how they were coded, see Supplementary Methods S1. Participants were instructed to collect their fecal samples at home using a validated kit and protocol (OMNIgene•GUT, DNAGenotek, Ottawa, CA, USA) and send them back to our laboratory for gut microbiota analyses [42]. We excluded participants with missing fecal samples, irritable bowel syndrome (IBS), >30% missing answers in the relevant online questionnaires, frequent antibiotics usage (frequent, sometimes, rarely, or never; 96.4% of participants answered never or rarely, one control participant reported frequent antibiotics usage) resulting in 77 participants with and 76 without ADHD. All analyses were performed in R (version 4.2.1 (R Core Team & Team, 2021)). This report follows the STORMS guidelines for reporting microbiome research where possible, see Supplementary Table S8 [43].

### 2.2. Statistical Analysis of Diet

#### 2.2.1. Dietary Patterns

To identify patterns of dietary habits, we applied the Exploratory Factor Analysis (EFA) (psych package [44]). We used the heterogeneous correlation matrix of questionnaire items to assess the factor loadings, as this accounts for the mixed ordinal categorical and continuous input from our dietary questionnaire [41,42], for more information on the EFA, see Supplementary Material Section S2.1.1.

#### 2.2.2. Diet–Behavior Associations

To investigate associations of the resulting diet factors with ADHD diagnosis we applied logistic regression due to the bivariate distribution. For associations of diet with reactive aggression, we chose nonparametric rank-based regression, which accounts for our nonnormal data reflecting observed reactive aggression scores in our sample (Rfit package [45]) across all participants, and corrected for ADHD diagnosis. 

### 2.3. Statistical Analysis of the Gut Microbiota

#### 2.3.1. Microbiota–Behavior Associations

The fecal sample wet-lab procedures, 16S rRNA sequencing of the V4 region, and data preprocessing are described in Supplementary Methods S2. We investigated associations of alpha diversity (e.g., observed number of amplicon-sequence-variants (ASVs), reflecting bacterial richness, Shannon diversity, which reflects the richness and evenness of the distribution, and Faith’s phylogenic diversity, which additionally accounts for the phylogenic relationships among the features) with reactive aggression using rank-based regression, and with ADHD diagnosis using logistic regression. We applied a permanova to test the associations of beta diversity (Aitchison distance on ASV level was applied, as it is considered to reflect the compositional data into the Euclidean space) with both behaviors (adonis2, vegan package [46]). For compositional analyses, the ASV table was aggregated to the genus level, and counts were center-log-ratio (CLR) transformed to account for the compositionality of the data. To reduce the number of tests, we further applied randomized Lasso feature selection (monaLisa package, 10% selection probability [47]). This method selected genera for subsequent association tests with reactive aggression and ADHD. We investigated associations between CLR-transformed abundances, which best reflect the compositional data, and reactive regression and ADHD diagnosis, with rank-based and logistic regression, respectively. We additionally analyzed abundance–behavior associations with a commonly used differential abundance analysis tool (ANOVA-like Differential Expression (ALDEx2) [48], as converging results across statistical tools are more likely to reflect true effects. For detailed descriptions of the statistical analyses and feature selection, see Supplementary Methods S3.

#### 2.3.2. Mediation Analysis of the Gut Microbiota on Diet and Behavior

**Figure 1:**
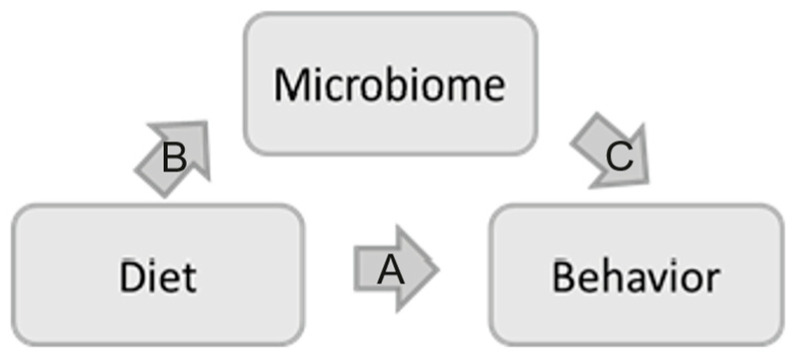
Diagram of potential mediating effects of the gut microbiota on the relationship between the diet factors and behavioral outcome measures such as ADHD diagnosis and reactive aggression.

To investigate the mediating effects of the gut microbiota on significant diet–behavior associations, we first identified genera that were potential mediators (using the mma package [49], default *p* < 0.1 for correlations with diet and behavior). Then, we applied a nonparametric mediation analysis of the diet–behavior relationship, as the normal distribution of our data was not given (mediation package [50], see Figure 1). This function of this package applies quantile regression to assess the direct effect (behavior–diet (A)) and indirect effects, accounted for by the mediator (diet–mediator (B) * mediator–behavior (C)) and estimates the significance of this mediation effect with a bootstrapping procedure [50].

All analyses on diet and the gut microbiota were corrected for age, sex, body mass index (BMI), and current smoking; the significance threshold was *p* < 0.05 and FDR-adjusted for relevant tests. We investigated associations with reactive aggression across all participants. Due to the case-control design of the study, significant associations with reactive aggression were additionally analyzed for associations with ADHD diagnosis.

## 3. Results

### 3.1. Demographic Description of the Sample

Demographic descriptions of the sample are presented in Table 1. A total of 77 participants with and 76 without ADHD were included. Both groups had comparable distributions of age, sex, and body mass index (BMI), but participants with ADHD more often reported current smoking and showed higher reactive aggression scores compared to neurotypical individuals.

### 3.2. Diet

#### 3.2.1. Dietary Patterns

**Figure 2:**
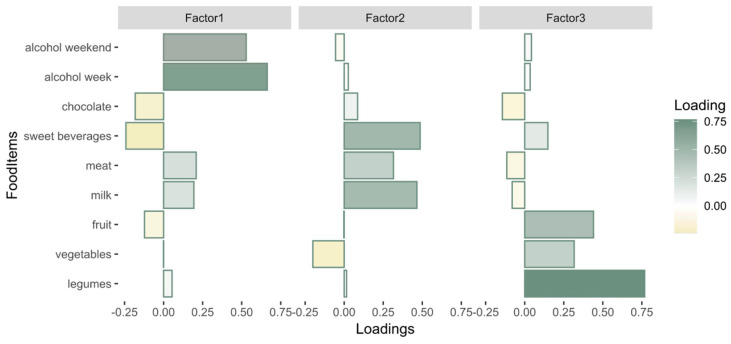
Factor loadings of the food items for the three diet factors suggested by EFA. Factor1, high-alcohol (**left**); Factor2, high-energy (**middle**); Factor3, high-fiber (**right**).

Parallel analysis in the EFA suggested a three-factor solution (Supplementary Figure S2). Factor1 was characterized by the high consumption of alcohol and meat and the low consumption of sweetened beverages and chocolate; we described this factor as high-alcohol; Factor2 was defined by the high consumption of sweetened beverages, milk, and meat and the low consumption of vegetables, resembling a high-energy diet similar to a Western diet; Factor3 showed the high consumption of legumes, fruits, and vegetables and the low consumption of meat, milk, and chocolate, describing a plant-based high-fiber diet (RMSE = 0.06, corrected for degrees of freedom). The factors were not correlated; see Supplementary Table S1 for further information. Figure 2 shows the loadings of each food item on the three factors.

#### 3.2.2. Diet–Behavior Associations

We found reactive aggression scores were associated with the high-energy diet Factor2. An ADHD diagnosis and the male sex were of relevance for this relationship; see Table 2 (top).

None of the dietary factors were associated with an ADHD diagnosis. The confounders, age, sex, and BMI, did not affect the outcome, while current smoking showed a positive association with an ADHD diagnosis; see Table 2 (bottom).

### 3.3. Microbiota

#### 3.3.1. Microbiota–Behavior Associations

Alpha diversity was not significantly associated with reactive aggression or an ADHD diagnosis. Current smoking was not a relevant predictor for reactive aggression, but age, sex, and BMI did show effects in both models; see Supplementary Results S2.1. Beta diversity was not associated with reactive aggression, but was with an ADHD diagnosis (F = 1.24, R2 = 0.008, p = 2.9 × 10^−02^); see Supplementary Results S2.2.

Our feature selection step selected nine genera for reactive aggression and nine genera for ADHD diagnosis, see Supplementary Results S3. The genera selected for reactive aggression scores and an ADHD diagnosis did not overlap. Eight out of nine selected genera were significantly associated with reactive aggression in the logistic regression, and ALDEx2 identified three converging results: *Eubacterium xylanophilum group*, *Lactobacillus*, and *Slackia*; see Supplementary Figure S6. *Lactobacillus* (*p*_FDR_ = 3.9 × 10^−02^) and *Slackia* (*p*_FDR_ = 9.0 × 10^−03^) remained significant after correction for an ADHD diagnosis but the *Eubacterium xylanophilum group* did not (*p*_FDR_ = 1.3 × 10^−01^); see Supplementary Figure S7 and Supplementary Table S4. All selected genera for ADHD were significantly associated with ADHD after FDR correction using logistic regression. Six of these genera (*Tyzzerella*, *RF39*, *Sutterella*, *uncultured 6*, *Eisenbergiella*, and *Eubacterium fissicatena group*) were additionally identified using ALDEx2; see Supplementary Figure S8 for relative abundance plots. Table 3 summarizes the results of the feature selection and differential abundance analysis, significant associations are visualized in the Supplementary Materials Sections S2.3.2 and S2.3.4.

#### 3.3.2. The Gut Microbiota as a Mediator of Diet and Behavior

Two genera, *Eubacterium nodatum group* and *Lachnospiraceae UCG 010*, were identified as potential mediators, based on suggested correlations (*p* < 0.1), with both reactive aggression and the high-energy diet factor (see Supplementary Table S5) [47]. These two genera were not previously tested for association with reactive aggression as they were not selected in the feature selection step. The genera associated with reactive aggression did not show a correlation with the high-energy diet Factor2. Mediation analyses of the two selected genera on the association of reactive aggression with the high-energy diet showed no significant mediation effect; see Supplementary Table S6 for a summary of the mediation analysis.

## 4. Discussion

To our knowledge, this is the first study investigating the role of diet and the gut microbiome in reactive aggression, together with the potential mediating effects of the gut microbiota on the relationships between diet, ADHD, and reactive aggression. We found a positive association between a high-energy dietary pattern and reactive aggression, and we observed gut microbial alterations in reactive aggression and ADHD. No mediation effects were seen.

### 4.1. Diet

We identified three dietary factors in our study population, resembling a high-alcohol, a high-energy, and a high-fiber dietary pattern. Our results resemble the dietary patterns from Shi and colleagues (2022) [51], who used a similar questionnaire in a healthy population sample. An ADHD diagnosis was not associated with any of these dietary patterns. While recent meta-analyses on dietary patterns in children with ADHD had reported an unhealthier “Western diet” with high caloric and low nutritional food intake [52], the few published studies on adults had inconsistent results [53,54,55]. The self-reported food intake measure in adults may introduce an increased bias towards a healthy diet compared to parent-reported food intake in children. However, the association of our dietary questionnaire with reactive aggression supports the potential to identify the altered eating patterns in adult self-report measures, suggesting a generally similar diet in adults with and without ADHD. Other factors, for example, the gut microbiota that could result in different bioavailability of nutrients between individuals with and without ADHD, have to be investigated. Reactive aggression was positively associated with the “high-energy” diet factor. This dietary factor consists of the high consumption of sweetened beverages, meat, and dairy, as well as the low consumption of vegetables, legumes, and fruit. In line with this pattern, a recent study investigating dietary patterns in aggressive men reported a protective effect of legumes, vegetables, and fruit for aggression; in contrast to our study, however, meat and milk also showed protective effects for aggressive symptoms [56]. Despite the lack of research on the relationship between reactive aggression and diet. This finding matches the literature on (emotion) regulation and negative effects [23,24]. Western diet, and sweetened foods and beverages in particular, have been associated with (chronic) pro-inflammatory processes (for a review see [57]), which might be relevant for altered brain functioning [58] and reactive aggression [16]. Interventions reducing sweetened foods/drinks and improving the nutritional value by introducing more vegetables are not only beneficial for overall and cardiometabolic health in clinical and nonclinical populations but may benefit individuals prone to reactive aggression. This result highlights the relevance of diet for mental health, informing not only clinical practice to integrate dietary interventions for the treatment of mental health issues but also public health policies to prevent adverse mental health outcomes.

### 4.2. Gut Microbiota

The associations between gut microbiota and an ADHD diagnosis were largely similar to our recent meta-analysis on this topic (N total = 617, Jakobi et al. (2023) [59]), in which IMpACT2-NL was included. While alpha diversity was not associated with reactive aggression, beta diversity showed significant differences between adults with and without ADHD. A higher abundance of *Eisenbergiella*, observed in adults with ADHD compared to neurotypical peers, was also seen in our recent meta-analysis. *Eisenbergiella* has been previously associated with pro-inflammatory processes and immune activation [60,61,62] and was shown to be enriched in psychiatric [63,64] and metabolic disorders such as gestational diabetes mellitus [65]. These associations might suggest a pathogenic role. Alternatively, the higher abundance could be a consequence/epiphenomenon of disadvantageous health outcomes.

Reactive aggression was neither associated with alpha nor beta diversity in this study. However, we identified several genera associated with reactive aggression. *Slackia* and Lactobacillus were associated with reactive aggression scores across participants and the *Eubacterium xylanophilum* group with lower scores. Studies reporting the effects of probiotic intervention including Lactobacillus on cognition and brain functioning during emotion processing [66,67], and enriched *Slackia* abundance in association with low female sex-hormone levels [68] support the potential involvement of these genera in biological pathways relevant for reactive aggression, but these results have to be replicated.

In this study, we found no overlap in selected genera for an ADHD diagnosis and reactive aggression. However, a lower abundance of the *Eubacterium xylanophilum* group, associated with high reactive aggression scores, was also associated with an ADHD diagnosis in our meta-analysis [59]; a small effect size may have led to the negative result in the current study. The *Eubacterium xylanophilum* group is a producer of short-chain-fatty acids (SCFAs) [69] from polyphenols (nutritional compounds of plant-based food items such as nuts, legumes, vegetables, and fruits) [70,71] and shows reduced abundance in women with gestational diabetes [65]. This bacterial genus may therefore have beneficial effects on immune functioning and inflammation [72]. While these findings of microbiota associations with reactive aggression and ADHD suggesting the inclusion of immune pathways are in line with recent immune-psychiatry frameworks, mechanistic studies including metatranscriptomics and metabolomics are need to shed light on the contribution of neuroendocrine and neurological pathways to such disorders and assess the clinical potential of interventions targeting inflammation, hormones, and neurological stimulation. 

### 4.3. Mediation

We identified two genera as potential mediators of the association between the “high-energy” diet factor and reactive aggression, *Lachnospiraceae* UCG 010 and the *Eubacterium nodatum* group. Both genera tended to be less abundant in individuals with higher reactive aggression scores and less abundant in relation to higher scores on the high-energy diet (low in vegetables). Indeed, these taxa have been shown to be enriched in plant-based diets [69,73,74]. The *Eubacterium nodatum* group is involved in inflammatory processes in different ways (SCFA producer [69], a pathogen in oral infections [75], and increased *Lachnospiraceae*). UCG 010 abundance was associated with lower cholesterol and alterations in tryptophane metabolism after cholesterol intervention using grape powder in healthy individuals [76]. While general health benefits suggest that these genera might mediate the positive effects of diet on behavior, causal mediation analysis showed no significant mediation effects. This could result from taxonomic clustering; at the genus level, several species and strains with various functional properties can be clustered together. Higher functional resolution (e.g., achieved by metagenomic sequencing to the species level, or clustering of sequences for functional pathways) could provide more meaningful associations and a more powerful way to investigate this question. Other than influencing the microbiome, diet might also affect behavior by directly acting on the enteric nervous system, which we did not investigate in the current study.

### 4.4. Strengths and Limitations

The current study shows strengths and limitations that should be considered when interpreting the results. This is the first study to investigate diet and the gut microbiome in reactive aggression and the first one to investigate potential mediations of gut microbiota on diet–reactive aggression and diet–ADHD associations. Focusing the analyses on European samples of adult ADHD in the case-control design reduces heterogeneity, however, these factors should be considered to hamper translation to other age groups, contexts, and populations. While we have highlighted the importance of including diet information in the study of mental health, detailed and reliable nutritional data can be difficult to obtain.

As in most nutritional studies, the dietary questionnaire implemented in this study was a self-report measure in which we traded acceptability by the participants of the study with reduced reliability compared to more laborious measures [72]. Our questionnaire had low-resolution answer options (“portions”, “pieces”, and “glasses”) and concentrated on a limited number of major food groups. Using a selection of food items that could be categorized as generally beneficial/healthy or disadvantageous/unhealthy, we described our dietary factors in terms of nutritional value. The limitations of the self-reports of diet in adults in this and other studies might hamper the detection of consistent effects of diet in adult ADHD (in contrast to studies in children with ADHD [20]) and related behaviors [77]. Despite the low resolution of our diet measure, we identified meaningful dietary patterns, replicating the results of other studies with similar questionnaires, and found a significant association of a ‘high-energy’ diet factor with reactive aggression scores, potentially highlighting the relevance of nutrition for healthy, situation-appropriate behavior.

On the side of strengths, the identification of food items and dietary patterns that ameliorate or aggravate reactive aggression, as provided in this study, and/or symptoms of ADHD are prerequisites for the development of targeted dietary interventions; an affordable treatment support with little side effects, benefitting individuals beyond the behavioral symptoms (e.g., overall health and metabolic comorbidities). While some nutritional intervention studies in ADHD have shown promising results in ADHD treatment support [22,78], more evidence for treatment success of targeted nutritional interventions is needed [78]. Higher resolution diet data, stemming from standardized food frequency questionnaires (FFQ, e.g., [56]), combined with randomized controlled trials for nutritional intervention studies should be applied to investigate the potential disadvantageous effects of high-energy diets and potential protective roles of food groups, e.g., PUFAs, for the emergence of reactive aggression and adult ADHD. Other confounders of diet and overall health should be considered when studying diet and the gut microbiome. Socio-economic status, education level, physical exercise, and sleep, for example, have been reported to influence dietary choices and overall health [74,75].

A final set of both strengths and limitations is related to microbiota research. While high interindividual differences in microbiota communities hamper the detection and estimation of effects, make power calculations on an individual feature level difficult, and may require larger samples, this study is the second-largest currently available study of gut microbiota–ADHD associations and the only one of gut microbiota–reactive aggression associations to date. Effect sizes in the zero-inflated data-masses of microbial sequencing data are expectedly small. For robust results, bigger study populations would be beneficial. Additionally, the measuring of gut-microbiota and the statistical methods applied to identify associations of the compositional data have been shown to often disregard the underlying distributions of the data, and their compositionality, and produce different results. We applied a threefold strategy to mitigate these limitations: Firstly, we reduced the statistical testing burden of uninformative genera by applying a feature selection step prior to differential abundance analysis; Secondly we corrected all analyses for common confounders; thirdly, we per-formed analyses using several state-of the art statistical tools (logistic regression, ALDEx2) and indices (observed ASVs, Shannon index, Faith’s phylogenic diversity) to identify con-verging results.

## 5. Conclusions

In the current study, we showed that diet and the gut microbiota play a role in reactive aggression and adult ADHD. If replicated, these results could help identify targets for nutritional interventions or microbiota-targeted pre-/probiotics as treatment support for reactive aggression, especially in the context of adult ADHD. While inflammatory processes might play a role in both reactive aggression and ADHD, the mechanisms at play in the interaction of diet, the gut microbiota, and these behaviors deserve more investigation. To do so, large studies with detailed dietary phenotyping are needed to robustly identify dietary and microbial signatures of ADHD or ADHD-related behaviors. Longitudinal studies and clinical interventions targeting diet and the gut microbiota (probiotics) may help unravel the causal relationships between diet, behavior, and the gut microbiome and introduce meaningful additions to clinical practice. In addition, the use of metagenomic sequencing, pathway analysis, and clustering based on functional capacities instead of phylogenetic relationships, as well as microbial culturing and basic research into gastrointestinal environments, are needed to characterize the mechanisms involved.

## Figures and Tables

**Table 1:** Demographic description of the sample.

	ADHD	Controls
N	77	76
Age in years, Mean (SD)	34.09 (10.37)	34.43 (12.9)
Sex, % male	42.86%	47.82%
BMI as kg/m^2^, Mean (SD)	24.90 (4.51)	24.82 (4.1)
Smoking,% current non-smokers	70.13%	90.91%
Stimulant medication, % current users	57.14%	0%
Reactive aggression ^1^, Mean (SD)	8.23 (4.04)	5.64 (3.2)
Number of inattentive symptoms ^2^, Mean (SD)	7.34 (1.90)	0.83 (1.2)
Number of hyperactive/impulsive symptoms ^2^, Mean (SD)	5.62 (2.22)	0.81 (1.1)

Demographic description of the sample consisting of participants with an ADHD diagnosis (ADHD) and without ADHD diagnosis (Controls). This table includes the mean and standard deviation of age, BMI, mean-centered diet scores, and reactive aggression scores as well as the percentage of current stimulant users, current non-smokers, and male participants. Antibiotic and probiotic usage were assessed on a scale of often, sometimes, rarely, or never: no participant used antibiotics frequently, and 7 participants used probiotics frequently. Statistical testing was performed using the Mann–Whitney test as well as the Chi-squared test for distribution-free comparisons of independent samples. SD = standard deviation. ^1^ Measured by the scores in the reactive aggression subscale of the RPQ [41], ^2^ Measured by the respective subscales of the DIVA questionnaire [40].

**Table 2:** Diet–behavior associations.

	Standard Estimate	Estimate	Std. Error	z-Value/ t-Value	*p*-Value	*p*-Value FDR
Reactive Aggression ~ Factor1 + Factor2 + Factor3 + age + sex + BMI + smoking + ADHD diagnosis						
Factor1	0.37	0.16	0.22	0.74	4.6 × 10^−01^	1
Factor2	0.82	0.51	0.19	2.73	7.0 × 10^−03^	4.22 × 10^−02^
Factor3	0.30	0.12	0.21	0.60	5.5 × 10^−01^	1
Age	0.06	0.00	0.02	0.11	9.1 × 10^−01^	n.a.
Sex	−0.76	−2.03	0.55	−3.72	2.8 × 10^−04^	n.a.
BMI	0.33	0.04	0.06	0.67	5.0 × 10^−01^	n.a.
Smoke	0.07	0.05	0.40	0.13	8.9 × 10^−01^	n.a.
ADHD diagnosis	0.75	2.22	0.55	4.04	8.4 × 10^−05^	5.85 × 10^−04^
ADHD Diagnosis ~ Factor1 + Factor2 + Factor3 + age + sex + BMI + smoking						
Factor1	−0.26	−0.11	0.15	−0.73	4.7 × 10^−01^	1
Factor2	−0.01	0.00	0.12	−0.03	9.7 × 10^−01^	1
Factor3	0.02	0.01	0.13	0.06	9.5 × 10^−01^	1
Age	0.02	0.00	0.01	0.04	9.7 × 10^−01^	n.a.
Sex	0.36	0.36	0.36	1.01	3.2 × 10^−01^	n.a.
BMI	0.02	0.00	0.04	0.05	9.6 × 10^−01^	n.a.
Smoke	1.13	0.87	0.27	3.21	1.6 × 10^−03^	n.a.

Results from the logistic regression analysis of dietary factors with ADHD diagnosis (bottom), and the rank-based regression with reactive aggression (top), corrected for age, sex, BMI, current smoking, and an ADHD diagnosis, showing standardized regression estimates, estimates, standard errors, z-statistic (for the nonparametric regression t-statistic), and *p*-values. FDR correction was applied to *p*-values of associations of the dietary factors with ADHD diagnosis or reactive aggression. Significant results of interest at an FDR-corrected threshold of *p* < 0.05 are highlighted in grey shading. n.a stands for not applicable.

**Table 3:** Microbiota–behavior associations.

Feature Selection		Logistic Regression/Rank-Based Regression					ALDEx2				
Genus	Sel. Prob.	Std. Error	Estimate	z	*p*	*p*_FDR_	Estimate	Std. Error	t	*p*	*p*_FDR_
Reactive Aggression											
*Lactobacillus*	0.27	0.11	0.28	2.54	1.2 × 10^−02^	1.7 × 10^−02^	−0.23	0.1	−2.42	1.9 × 10^−02^	5.2 × 10^−02^
*Slackia*	0.35	0.08	0.24	2.88	4.5 × 10^−03^	1.4 × 10^−02^	−0.32	0.13	−2.52	1.7 × 10^−02^	5.2 × 10^−02^
Eubacterium xylanophilum group *	0.11	0.12	−0.26	−2.23	2.7 × 10^−02^	2.9 × 10^−02^	0.21	0.1	2.23	3.3 × 10^−02^	5.3 × 10^−02^
*Dialister*	0.11	0.07	−0.16	−2.28	2.4 × 10^−02^	2.9 × 10^−02^	0.28	0.15	1.95	5.8 × 10^−02^	9.1 × 10^−02^
*Succiniclasticum*	0.19	0.15	0.33	2.25	2.6 × 10^−02^	2.9 × 10^−02^	−0.24	0.1	−2.46	4.4 × 10^−02^	9.9 × 10^−02^
*Allhorhizobium Neorhizobium Pararhizobium Rhizobium*	0.26	0.38	1.14	3.03	2.9 × 10^−03^	1.4 × 10^−02^	−0.12	0.08	−1.53	2.3 × 10^−01^	2.5 × 10^−01^
*Murdochiella*	0.21	0.35	0.91	2.6	1.0 × 10^−02^	1.6 × 10^−02^	−0.11	0.08	−1.51	2.3 × 10^−01^	2.5 × 10^−01^
*Lachnospiraceae*	0.13	0.22	0.51	2.31	2.2 × 10^−02^	2.9 × 10^−02^	−0.11	0.09	−1.29	2.7 × 10^−01^	2.8 × 10^−01^
*Atopobium*	0.11	0.26	−0.18	−0.69	4.9 × 10^−01^	4.9 × 10^−01^	0.09	0.08	1.16	3.3 × 10^−01^	3.4 × 10^−01^
ADHD Diagnosis											
*Tyzzerella*	0.59	0.06	0.22	3.82	1.3 × 10^−04^	2.4 × 10^−03^	−3.6	0.99	−3.65	9.7 × 10^−04^	9.4 × 10^−03^
*RF39*	0.25	0.06	−0.19	−3.39	7.1 × 10^−04^	6.4 × 10^−03^	3.13	0.9	3.49	1.2 × 10^−03^	9.4 × 10^−03^
*Eubacterium fissicatena group*	0.11	0.11	0.28	2.55	1.1 × 10^−02^	1.6 × 10^−02^	−1.48	0.59	−2.53	2.4 × 10^−02^	5.0 × 10^−02^
*Sutterella*	0.19	0.08	−0.24	−2.85	4.4 × 10^−03^	1.4 × 10^−02^	1.75	0.66	2.66	9.3 × 10^−03^	5.2 × 10^−02^
*uncultured.6*	0.15	0.06	−0.18	−2.78	5.4 × 10^−03^	1.4 × 10^−02^	2.3	0.9	2.57	2.1 × 10^−02^	5.2 × 10^−02^
*Eisenbergiella*	0.12	0.09	0.25	2.77	5.6 × 10^−03^	1.4 × 10^−02^	−1.79	0.67	−2.67	1.6 × 10^−02^	5.2 × 10^−02^
*Ruminiclostridium*	0.15	0.11	0.3	2.63	8.7 × 10^−03^	1.6 × 10^−02^	−1.47	0.71	−2.07	9.3 × 10^−02^	1.1 × 10^−01^
*Caulobacter*	0.11	0.16	0.43	2.64	8.3 × 10^−03^	1.6 × 10^−02^	−0.97	0.56	−1.74	1.4 × 10^−01^	1.5 × 10^−01^
*Sanguibacteroides*	0.16	0.15	−0.4	−2.58	9.8 × 10^−03^	1.6 × 10^−02^	1.25	0.65	1.93	1.3 × 10^−01^	2.1 × 10^−01^

Summary of the results of the feature selection, namely selected genera and selection probability, and the subsequent differential abundance analyses of ADHD Diagnosis (top) and reactive aggression (bottom) with two statistical approaches, logistic regression (left) and ALDEx2 (right), Estimate, standard error, z/t value, raw and FDR adjusted *p*-value corrected for all tests (18 for logistic regression, 36 when repeating the analyses in ALDEx2). Convergent results, significant in both and FDR-corrected in at least one method are highlighted in grey. * *Eubacterium xylanophilum* group did not remain significant after correction for ADHD diagnosis (*p*_FDR_ = 1.3 × 10^−01^).

## Data Availability

The anonymized participant data and raw sequencing data supporting the findings of this study is located at the Radboud Data Repository (https://doi.org/10.34973/nwfn-ms80). Due to the informed consent statement our participants signed, the data can be made available upon reasonable request to the authors.

## Supplementary Materials

The following supporting information can be downloaded at https://www.mdpi.com/article/10.3390/nu16142174/s1. Supplementary information about the used questionnaires, microbiota pre-processing and statistical analyses, exploratory factor analyses used to determine dietary patterns, visualizations and results supplemental to the microbiota-behavior association, post-hoc sensitivity analysesanalyses as well as the STORMS checklist. References [79,80,81,82] are cited in Supplementary Materials.
