# Supplementary material for: The Role of Diet and the Gut Microbiota in Reactive Aggression and Adult ADHD—An Exploratory Analysis
Source: Nutrients. 2024 Jul 9;16(14):2174. doi: 10.3390/nu16142174 (PMC11279949; doi:10.3390/nu16142174)
Supplement: Supplementary file 1 [file nutrients-16-02174-s001.zip › nutrients-3034287-SI.pdf]

# Supplementary Material

## 1 Supplementary Methods

### 1.1 Description of the questionnaires

The reactive-proactive questionnaire (RPQ) is a 23-item self-report, validated questionnaire inquiring 11 example sentences of reactive and 12 of proactive aggression [1]. The questions are rated by the participants based on how often they have experienced the situation/feeling or acted in the way that the example sentence describes in a scale of 0 (never), 1 (sometimes), and 2 (often). For each subscale, the sum of the points over the subscale-relevant questions represent the subscale score, where no cutoff was applied. We focus on the reactive aggression questionnaire as this behavior is particularly associated with ADHD and represents a severe form or emotion dysregulation.

The semiquantitative food questionnaire assesses the frequency (never, monthly, weekly, daily) of eight food items and beverages (meat, fruit, vegetables, legumes, chocolate, milk, sugary drinks, alcohol), Supplementary Figure 1 is a copy of this questionnaire in Dutch. The amount per indicated frequency was assessed (in pieces, portions, glasses, or bars) and normalized to weekly quantities.

### 1.2 Microbiota preprocessing

#### 1.2.1 Fecal sample processing

The fecal samples were received in our laboratory, aliquoted into 1.5 ml Eppendorf tubes, and stored in -80˚C. Further processing was done by Baseclear B.V by aliquoting 150 mg feces, isolating and purifying DNA using a bead-beating procedure with the ZymoBIOMICS DNA 96 MagBead kit in conjunction with Kingfisher. The V4 region of 16S ribosomal RNA (rRNA) gene was targeted for sequencing. Amplicons were built using the primers 515-F: TCGTCGGCAGCGTCAGATGTGTATAAGACAGGTGYCAGCMGCCGCGGTAA, 806Rb: GTCTCGTGGGCTCGGAGATGTGTATAAGACAGAGG-GACTACNVGGGTWTCTAAT using the Phusion High-Fidelity PCR Master Mix with an HF Buffer (Thermo Fisher Scientific). The Illumina NovaSeq 6000 platform with the Novaseq 6000 SP reagent kit v1 with 500 cycles (paired-end, 250 bp) was used. Reads were demultiplexed, filtered, and adapter sequences and control signals were removed [2]. The total number of reads was 126.015.919 across all samples (median per sample = 781. 345, range 288.695 – 1.204.337) with a median phred score of Q > 30 for all samples, indicating 99.9% sequencing accuracy.

# SECTIE C : VOEDING

In deze sectie vragen wij u om vragen te beantwoorden over **uw eetgewoontes en voedingspatronen.** Al de informatie die wij van u ontvangen zal vertrouwelijk blijven en dusdanig behandeld worden.

*** () Bent u vegetariër (geen vlees) of veganist (geen vlees en andere dierlijke producten, zoals kaas, melk, eieren, …)?**

Kies één van de volgende mogelijkheden:

- ○ Nee
- ○ Ja
- ○ Weet ik niet

*** () Hoe vaak eet u vlees? Dit mag alle soorten vlees zijn.**

**Beantwoord deze vraag alleen als aan de volgende voorwaarden is voldaan:**
VOE1 != 'Y'

Kies één van de volgende mogelijkheden:

- ○ Dagelijks
- ○ Wekelijks
- ○ Maandelijks
- ○ Weet ik niet

*** () Hoe vaak eet u fruit? Dit mag zowel vers fruit, als ingevroren, als in blik zijn.**

Kies één van de volgende mogelijkheden:

- ○ Dagelijks
- ○ Wekelijks
- ○ Maandelijks
- ○ Nooit
- ○ Weet ik niet

*** () Hoe vaak eet u bonen, erwten, kolen (bloemkool, savooikool, witte kool, …), broccoli en andere vezelbevattende groenten?**

Kies één van de volgende mogelijkheden:

- ○ Dagelijks
- ○ Wekelijks
- ○ Maandelijks
- ○ Nooit
- ○ Weet ik niet

*** () Aantal porties [per aangegeven tijdseenheid]:**

**Beantwoord deze vraag alleen als aan de volgende voorwaarden is voldaan:**
VOE5 == '0' || VOE5 == 1 || VOE5 == 2

In dit veld mogen alleen cijfers ingevoerd worden.

Vul uw antwoord hier in:

- [ ]

*** () Hoe vaak gebruikt u suikerbevattende dranken? (koud: Coca Cola, Sprite, Fanta, Ice-tea, Nestea, energiedrankjes zoals Red Bull, …) (warm: cappuccino, koffie met suiker, lattes, …)**

Kies één van de volgende mogelijkheden:

- ○ Dagelijks
- ○ Wekelijks
- ○ Maandelijks
- ○ Nooit
- ○ Weet ik niet

*** () Hoeveel chocolade eet u per maand?**

Kies één van de volgende mogelijkheden:

- ○ Ik eet geen chocolade
- ○ 0 -1 reep (a 200 gram)
- ○ 1 tot 5 repen (a 200 gram)
- ○ 5 - 10 repen (a 200 gram)
- ○ Meer dan 10 repen (a 200 gram)
- ○ Weet ik niet

*** () Is dit voornamelijk:**

**Beantwoord deze vraag alleen als aan de volgende voorwaarden is voldaan:**
VOE9 > 0

Kies één van de volgende mogelijkheden:

- ○ Puur
- ○ Melk
- ○ Wit

*** () Aantal stuks:**

**Beantwoord deze vraag alleen als aan de volgende voorwaarden is voldaan:**
VOE3 == '0' || VOE3 == 1 || VOE3 == 2

In dit veld mogen alleen cijfers ingevoerd worden.

Vul uw antwoord hier in:

- [ ]

*** () Hoe vaak eet u groenten?**

Kies één van de volgende mogelijkheden:

- ○ Dagelijks
- ○ Wekelijks
- ○ Maandelijks
- ○ Nooit
- ○ Weet ik niet

*** () Aantal porties [per aangegeven tijdseenheid]:**

**Beantwoord deze vraag alleen als aan de volgende voorwaarden is voldaan:**
VOE4 == '0' || VOE4 == 1 || VOE4 == 2

In dit veld mogen alleen cijfers ingevoerd worden.

Vul uw antwoord hier in:

- [ ]

*** () Aantal glazen/koppen:**

**Beantwoord deze vraag alleen als aan de volgende voorwaarden is voldaan:**
VOE6 == '0' || VOE6 == 1 || VOE6 == 2

In dit veld mogen alleen cijfers ingevoerd worden.

Vul uw antwoord hier in:

- [ ]

*** () Hoeveel alcohol gebruikt u doordeweeks per dag (wijn, bier, … ; geen sterke dranken)?**

Kies één van de volgende mogelijkheden:

- ○ 0 glazen per dag
- ○ 1 - 2 glazen per dag
- ○ 3 - 4 glazen per dag
- ○ 5 -6 glazen per dag
- ○ > 6 glazen per dag
- ○ Weet ik niet

*** () Hoeveel alcohol gebruikt u in het weekend per dag (wijn, bier, … ; geen sterke dranken)?**

Kies één van de volgende mogelijkheden:

- ○ 0 glazen per dag
- ○ 1 - 2 glazen per dag
- ○ 3 - 4 glazen per dag
- ○ 5 -6 glazen per dag
- ○ > 6 glazen per dag
- ○ Weet ik niet

*** () Hoeveel melk drinkt u per week?**

Kies één van de volgende mogelijkheden:

- ○ Geen
- ○ 1 -2 bekers per dag (a 200 ml)
- ○ 2 - 4 bekers per dag (a 200 ml)
- ○ Meer

*** () Is deze melk:**

**Beantwoord deze vraag alleen als aan de volgende voorwaarden is voldaan:**
VOE10 > 0

Kies één van de volgende mogelijkheden:

- ○ Gepasteuriseerd
- ○ Lang houdbaar

***Supplementary Figure 1.*** *Semiquantitative questionnaire on eating habits, copy of the Dutch original version.*

### 1.2.2 Preprocessing of sequencing data

Raw sequences were demultiplexed and denoised in DADA2 (QIIME2, [3]). The resulting 14.408 Amplicon Sequence Variants (ASVs) were classified (naive Bayes classifier pre-trained on the SILVA database 138 for the V3/V4 Region, https://docs.qiime2.org/2022.2/data-resources/). We filtered bacterial DNA (10.428 bacteria over 153 samples) and analyzed alpha and beta diversity on bacterial ASV level. For compositional analysis and the feature selection step in which we determined the taxa to investigate, we aggregated the sequences to the genus level (resulting in 624 genera) and applied a prevalence threshold of 10% - reducing the number of tests and increasing comparability between studies – (resulting in 240 genera) and a center-log-ratio (CLR) transformation to account for bias of compositionality and sequencing [4, 5].

## 1.3 Microbiota-behavior analyses

### 1.3.1 Alpha and beta diversity

We assessed three indices of alpha diversity: 1) the number of observed ASVs describes the richness; 2) the Shannon index combines information of the richness of identified taxa as well as the evenness of their distribution within a sample; 3) Faith's phylogenic diversity further integrates the phylogenic relationship between the taxa (*microbiome* package [6]). We applied rank-based regression models [7] to investigate associations of reactive aggression, and we applied logistic regression models to identify associations of ADHD diagnosis with alpha diversity indices. To investigate associations of both behaviors with beta diversity, we applied principal coordinate analysis on the CLR-transformed ASV counts (Aitchison distance) using two separate permanova models (*vegan* package [8]) for ADHD diagnosis and reactive aggression, respectively. All models were corrected for age, sex, BMI, and current smoking.

### 1.3.2 Feature selection and differential abundance analysis

To reduce the number of tests in compositional analyses, we first applied a feature selection step on the genus level data using random Lasso stability selection (*monaLisa* package, [9]). Randomized Lasso selection is based on stability rather than effect size. In a random subsample of n/2, the respective behavior was regressed against all genera in a lasso-penalized regression, and the procedure was repeated for 999 subsamples (the source code was adapted for logistic regression for the selection based on ADHD diagnosis). The selection probability was calculated as the number of permutations in which a genus was selected (i.e., $\beta \neq 0$) divided by the total number of permutations. Due to the high interindividual variability and a relatively small sample of 153 participants, this permutation on subsamples of zero-inflated data will lead to expectedly small stability and selection probabilities per genus. We plotted the stability paths over all permutations and genera, based on those plots we decided on the lenient selection probability threshold for both behaviors of $\geq$ 10%, to only test

associations of those genera with a stability path deviating from the center of mass (see Supplementary Figure 4). On these genera we performed univariate association tests with the respective outcome measures (logistic regression for ADHD diagnosis and rank-based regression for reactive aggression scores). In addition, we performed differential abundance analysis with ALDEx2, a commonly applied tool that uses CLR transformation and dirichlet sampling prior to statistical testing, which produced the most reproducible results across datasets and tools in a recent comparison by Nearing and colleagues (2022). We corrected for age, sex, BMI, and current smoking in all models. P-values were FDR-corrected for all employed models over both behavioral outcomes. We further investigated effects of ADHD diagnosis on significant associations with reactive aggression, by adding ADHD diagnosis to the model across all participants and effects of current use of ADHD medication on significant associations with ADHD diagnosis in participants with ADHD only.

# 2 Supplementary Results

## 2.1 Diet

### 2.1.1 Diet Exploratory Factor Analysis

EFA is a method that instead of testing a pre-specified theoretical model, identifies the structure of a set of variables. While EFA may produce biased results in small sample sizes, we chose EFA, because of its ability to identify underlying factors that influence observed variables. To identify the optimal number of factors to extract, we applied parallel analysis, see Figure 2 for the resulting scree plot. After generating the heterogeneous correlation matrix, we extracted the factor loadings and error variances based on their maximum likelihood to have produced this correlation matrix. The resulting correlations between the factors resulting from the EFA are low, Factor1 describes a high-alcohol diet, Factor2 describes a high-energy diet, Factor3 describes a high-fiber diet, see Table 1. Bartlett scores were extracted and mean-centered, to assess how each individual scores on a particular diet factor. In this method, the factor scores of the individual highly correlate with one factor but not the others, considering them less biased.

Table 1. Factor correlations

| | Factor1 | Factor2 | Factor3 |
|---|---|---|---|
| Factor1 | 1 | -0.03 | 0.08 |
| Factor2 | -0.03 | 1 | -0.07 |
| Factor3 | 0.08 | -0.07 | 1 |

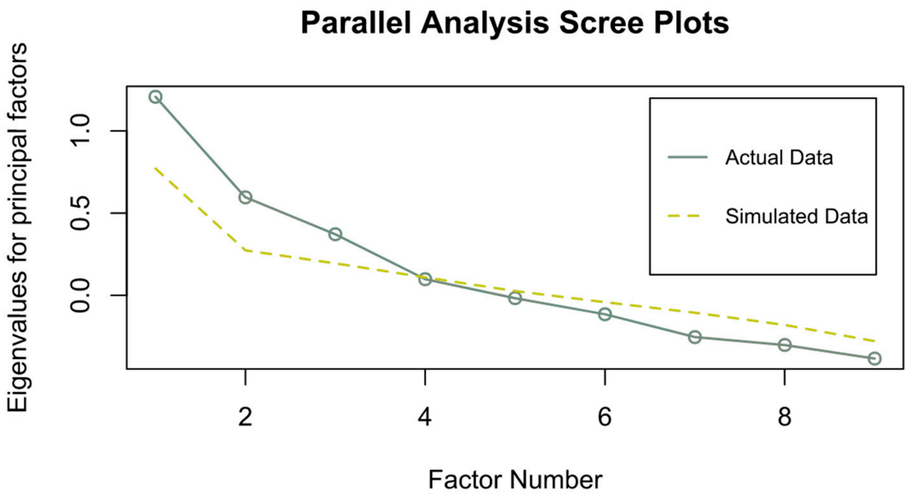


***Figure 2.*** *Scree plot, results of the parallel analysis. Actual data explains more variance compared to random simulated data between 1 and 3 factors, suggesting a 3-factor solution.*

### 2.1.2 Diet - behavior associations

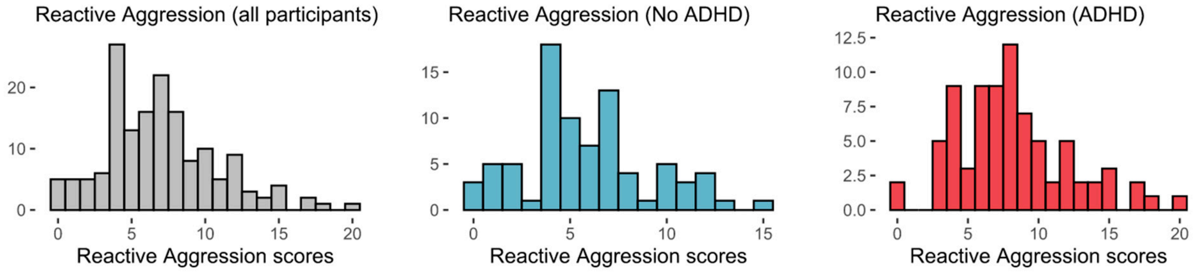


***Figure 3.*** *Distribution of reactive aggression scores across participants with and without ADHD.*

## 2.2 Microbiome - behavior associations

### 2.2.1 Alpha Diversity

Alpha diversity (observed ASVs, Shannon index and Faith's Phylogenic Diversity) was neither associated with reactive aggression, see Supplementary Table 2 (top), nor with ADHD diagnosis, see Supplementary Table 2 (bottom). However, as previously shown, age, sex, and BMI in both models. Current smoking was not associated.

Table 2. Associations of alpha diversity with ADHD and reactive aggression

| | Observed ASVs | | | | Shannon Diversity | | | | Faiths Phylogenic Diversity | | | |
|---|---|---|---|---|---|---|---|---|---|---|---|---|
| | Estimate | Std. Error | t-value/ z-value | *p*-value | Estimate | Std. Error | t-value/ z-value | *p*-value | Estimate | Std. Error | t-value/ z-value | *p*-value |
| **Reactive Aggression scores ~ alpha** + age + sex + BMI + smoking + ADHD Diagnosis | | | | | | | | | | | | |
| Alpha | 0.00 | 0.00 | -0.19 | 6.8E-01 | 0.02 | 0.60 | 0.04 | 9.7E-01 | 0.02 | 0.07 | 0.32 | 7.5E-01 |
| Age | 0.01 | 0.02 | 0.52 | 5.1E-01 | 0.01 | 0.02 | 0.45 | 6.5E-01 | 0.00 | 0.03 | 0.35 | 7.2E-01 |
| Sex | -2.27 | 0.52 | -4.37 | 5.5E-04 | -2.30 | 0.55 | -4.33 | 4.2E-05 | -2.29 | 0.54 | -4.28 | 3.3E-05 |
| BMI | 0.05 | 0.06 | 0.89 | 3.3E-01 | 0.05 | 0.06 | 0.89 | 3.7E-01 | 0.06 | 0.06 | 0.95 | 3.4E-01 |
| Smoking | 0.19 | 0.37 | 0.53 | 8.1E-02 | 0.19 | 0.38 | 0.52 | 6.0E-01 | 0.37 | 0.38 | 0.51 | 6.1E-02 |
| ADHD Diagnosis | 2.48 | 0.52 | 4.69 | 6.1E-06 | 0.53 | 0.54 | 4.61 | 8.5E-06 | 2.51 | 0.53 | 4.66 | 7.1E-06 |
| **ADHD Diagnosis ~ alpha** + age + sex + BMI + smoking | | | | | | | | | | | | |
| Alpha | 0.00 | 0.00 | -1.65 | 9.8E-02 | -0.30 | 0.39 | -0.78 | 4.4E-01 | -0.08 | 0.04 | -1.93 | 5.3E-02 |
| Age | 0.01 | 0.02 | 0.80 | 4.2E-01 | 0.00 | 0.02 | 0.29 | 7.7E-01 | 0.01 | 0.02 | 0.91 | 3.6E-01 |
| Sex | 0.46 | 0.35 | 1.32 | 1.9E-01 | 0.44 | 0.36 | 1.22 | 2.2E-01 | 0.51 | 0.36 | 1.44 | 1.5E-01 |
| BMI | -0.01 | 0.04 | -0.23 | 8.2E-01 | -0.01 | 0.04 | -0.13 | 9.0E-01 | -0.01 | 0.04 | -0.25 | 8.1E-01 |
| Smoking | 0.83 | 0.26 | 3.23 | 1.2E-03 | 0.82 | 0.26 | 3.19 | 1.4E-03 | 0.83 | 0.26 | 3.24 | 1.2E-03 |

*Results of the regression analysis of alpha diversity indices with reactive aggression scores (top) and ADHD diagnosis (bottom), corrected for age, sex, BMI, smoking, and ADHD diagnosis, showing estimates, standard errors, t / z -statistic and p-values.*

## 2.2 Beta Diversity

Beta diversity was associated with age and sex in both models, but not with reactive aggression scores. ADHD diagnosis was associated with beta diversity before FDR correction, but the effect did not survive the correction, see Supplementary Table 3 for all results and Supplementary Figure 4 for the supervised Canonical analysis of principal coordinates (CAP) plot of beta diversity for ADHD diagnosis.

Table 3. Associations of beta diversity with ADHD and reactive aggression

| | SumOfSqs | $R^2$ | F | *p*-value |
|---|---|---|---|---|
| **Beta ~ Reactive Aggression** + age + sex + BMI + smoking + ADHD Diagnosis | | | | |
| RPQ Reactive Aggression | 6367 | 0.007 | 1.01 | 3.8E-01 |
| Age | 16853 | 0.017 | 2.69 | 1.0E-03 |
| Sex | 9080 | 0.009 | 1.45 | 6.0E-03 |
| BMI | 7423 | 0.008 | 1.18 | 7.9E-02 |
| Smoking | 7411 | 0.007 | 1.14 | 1.2E-01 |
| ADHD diagnosis | 8054 | 0.008 | 1.28 | 2.4E-02 |
| **Beta ~ ADHD Diagnosis** + age + sex + BMI + smoking | | | | |
| ADHD | 7825472 | 0.008 | 1.25 | 2.9E-02 |
| Age | 16914176 | 0.017 | 2.69 | 1.0E-03 |
| Sex | 9053954 | 0.009 | 1.44 | 8.0E-03 |
| BMI | 7454836 | 0.008 | 1.19 | 6.9E-02 |
| Smoking | 7143587 | 0.007 | 1.14 | 1.1E-01 |

*Results of the permanova with 999 permutations of Aitchison distance between samples with and without ADHD (top) and reactive aggression scores (bottom), corrected for age, sex, SMI, smoking, and ADHD medication, where appropriate. We report sums of squares (SumOfSqs), R-squared, F statistic, and p values. Relevant p-values for the effects of interest were FDR-corrected over both models. Significant results are highlighted.*

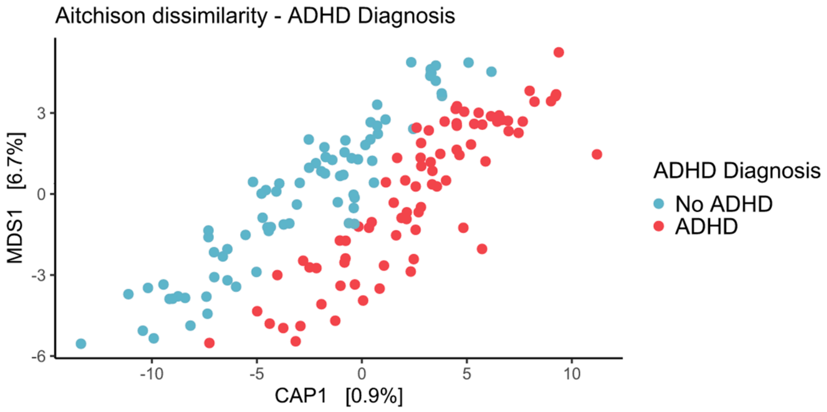


*Figure 4. CAP plot supervised for ADHD diagnosis. Individuals without ADHD are marked red. Individuals with ADHD are marked in blue.*

## 2.3 Feature selection and Differential abundance analysis

### 2.3.1 Feature selection stability path plots behavior

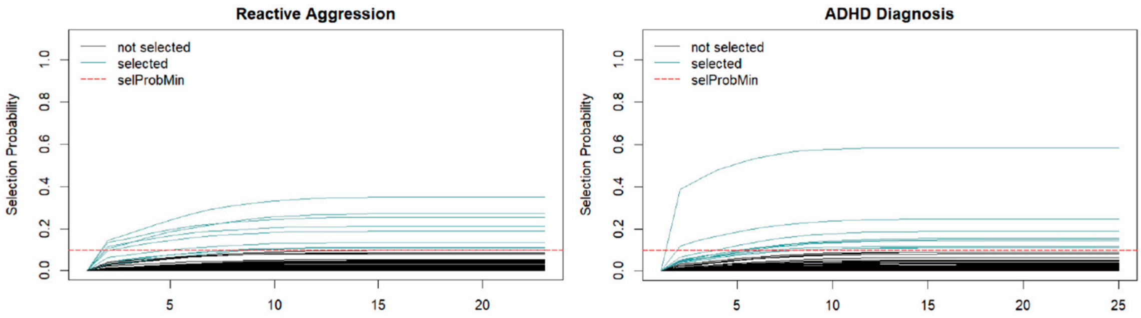


*Figure 5. Stability path plots from random Lasso stability selection for ADHD diagnosis (left) and reactive aggression scores (right). The red line marks the chosen selection probability of 10% over both behaviors, selecting genera whose stability path deviated visually from the majority of results close to zero over all outcomes.*

### 2.3.2 Reactive Aggression – Composition: relative abundance plots of significant genera

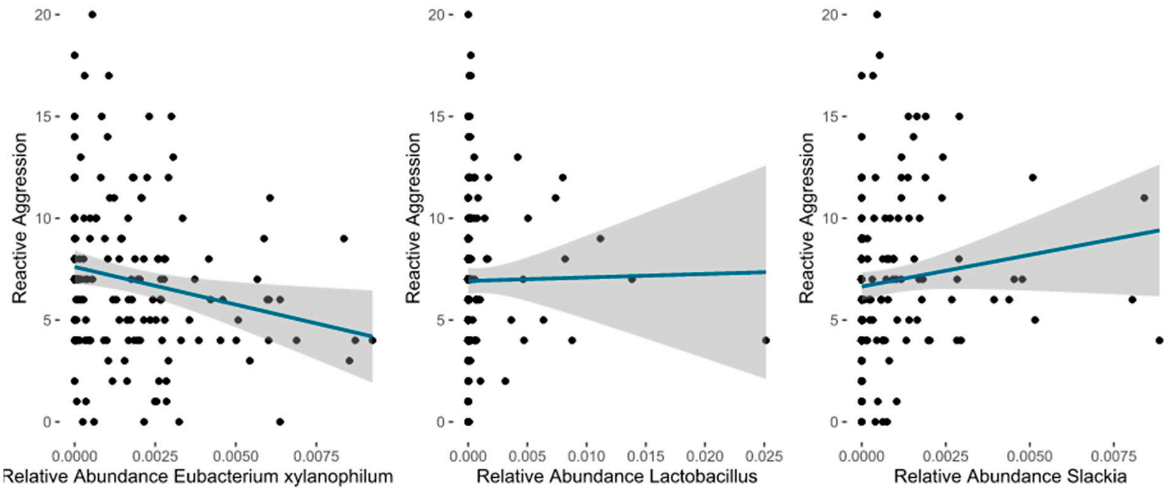


***Figure 6.*** *Relative abundance of the genera that were significantly associated with reactive aggression across all participants and Eubacterium xylanophilum group in participants with (red) and without ADHD (blue), plotted by reactive aggression scores.*

### 2.3.3 Reactive Aggression – Composition: correction for ADHD Diagnosis

**Table 4.** Correction for ADHD Diagnosis

| | Estimate | Std. Error | z-value / t-value | *p*-value |
|---|---|---|---|---|
| Reactive Aggression ~ genus + age + sex + bmi + ADHD Diagnosis | | | | |
| Lactobacillus | 0.23 | 0.10 | 2.23 | 2.7E-02 |
| Slackia | 0.23 | 0.08 | 2.95 | 3.6E-03 |
| Eubacterium xylanophilum group | -0.15 | 0.11 | -1.49 | 1.4E-01 |

| Analysis in individuals with ADHD and without ADHD separately | | | | |
|---|---|---|---|---|
| Eubacterium xylanophilum group ADHD | -0.09 | 0.04 | -1.87 | 6.5E-02 |
| Eubacterium xylanophilum group No ADHD | -0.06 | 0.05 | -1.21 | 2.2E-01 |

*Results of significant associations with reactive aggression, corrected for ADHD diagnosis. Eubacterium xylanophilum group was significantly associated with ADHD Diagnosis, the association with reactive aggression was not significant after correction for ADHD Diagnosis. Within-group analysis shows sub-threshold associations of Eubacterium xylanophilum group with reactive aggression in the ADHD group.*

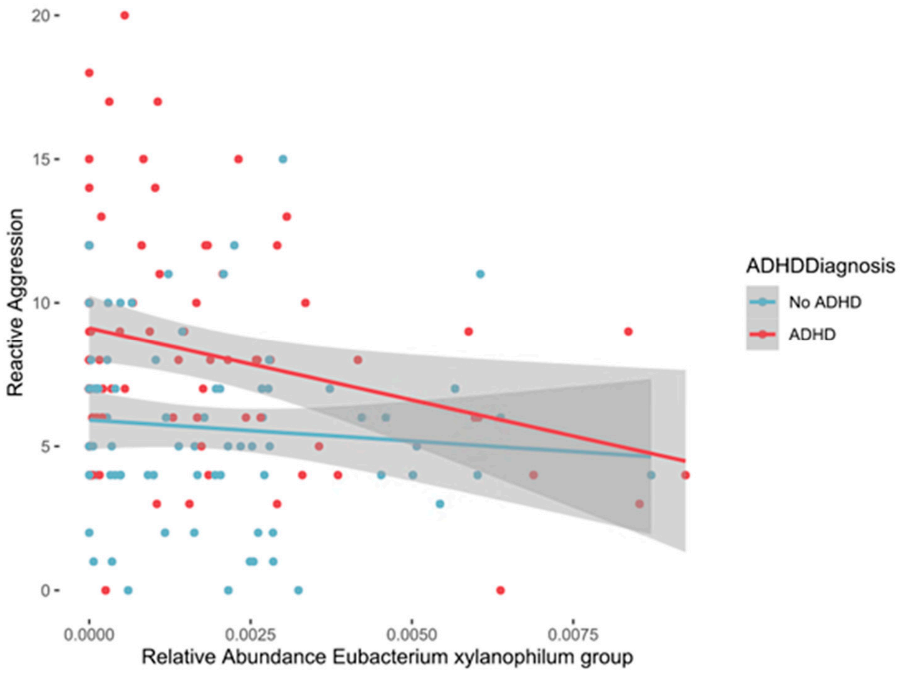


***Figure 7.*** *Relative abundance of Eubacterium xylanophilum group in participants with (red) and without ADHD (blue), plotted by reactive aggression scores.*

### 2.3.4 ADHD diagnosis – Composition: relative abundance plots of significant genera

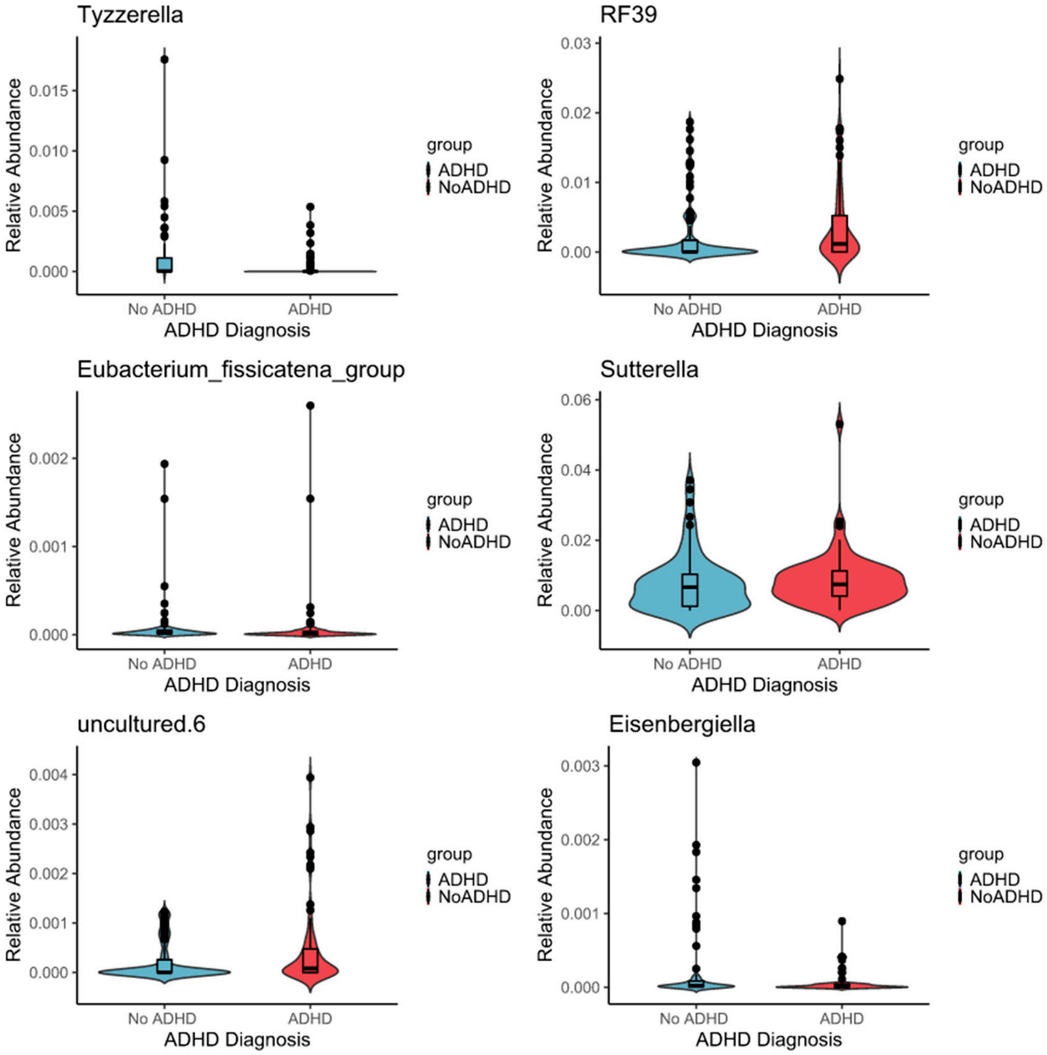


*Figure 8. Relative abundance plots of the genera that were significantly different between adults with and without ADHD across logistic regression and ALDEx2.*

### Table 5. Medication effects in participants with ADHD

| | Estimate | Std. Error | z-value | p-value |
|---|---|---|---|---|
| ADHD medication ~ genus + age + sex + bmi + smoking | | | | |
| *Tyzzerella* | -0.02 | 0.07 | -0.32 | 7.48E-01 |
| *Uncultured.6* | 0.14 | 0.10 | 1.38 | 1.67E-01 |
| *Eubacterium fissicatena group* | -0.08 | 0.16 | -0.45 | 6.50E-01 |
| *RF39* | 0.08 | 0.08 | 1.03 | 3.05E-01 |
| *Eisenbergiella* | 0.01 | 0.11 | -0.06 | 9.47E-01 |

| | | | | |
|---|---|---|---|---|
| *Sutterella* | -0.04 | 0.08 | -0.45 | 6.50E-01 |

*Associations of the group-different genera with current usage of ADHD medication in participants with ADHD diagnosis only.*

## 2.4 Mediation analysis

### Table 6. Selection of potential mediators

| | *High-energy Diet Factor* | | *Reactive Aggression* | |
|---|---|---|---|---|
| Genus | Rho | *p-value* | Rho | *p-value* |
| *Eubacterium nodatum group* | -0.15 | 5.66 E-02 | -0.13 | 9.25 E-02 |
| *Lachnospiraceae UCG 010* | -0.17 | 3.65 E-02 | -0.16 | 4.51 E-02 |

*P-values and rho from the identification step as potential mediator. Potential mediators are identified by p< .1 for both, independent variable (high-energy diet factor) and dependent variable (Reactive Aggression) (default setting).*

### Table 7. Results of the mediation analysis

| Mediation | Estimate | 95% CI lower | 95% CI upper | *p*-value |
|---|---|---|---|---|
| Eubacterium_nodatum_group | | | | |
| ACME | 0.07 | -0.01 | 0.15 | 6.6 E-2 |
| ADE | 0.74 | 0.25 | 1.15 | 4.0 E-3 |
| Total effect | 0.81 | 0.34 | 1.15 | 4.0 E-3 |
| Proportion Mediated | 0.08 | -0.02 | 0.30 | NA |
| Lachnospiraceae_UCG_010 | | | | |
| ACME (average) | 0.03 | -0.03 | 0.13 | 3.3 E-1 |
| ADE (average) | 0.84 | 0.23 | 1.24 | 1.2 E-2 |
| Total effect | 0.86 | 0.39 | 1.28 | 8.0 E-3 |
| Proportion Mediated | 0.034 | -0.06 | 0.22 | NA |

*Results of the mediation analyses of Eubacterium nodatum group (top) and Lachnospiraceae UCG 010 (bottom) on the association of the* ***high-energy*** *diet factor with reactive aggression scores. Estimates, p values, lower and upper boundaries of the confidence intervals for the Average Causal Mediated Effect (ACME), Average Direct Effect (ADE), total effect, and the proportion of the effect that was mediated are presented.*

## 3 STORMS checklist

### Table 8. STORMS guidelines for reporting microbiome research

| # | Item | Recommendation | Item Source | Additional Guidance | Yes/No /NA | Comments or location in manuscript |
|---|---|---|---|---|---|---|
| Abstract | | | | | | |
| 1.0 | Structured or Unstructured Abstract | Abstract should include information on background, methods, results, and conclusions in structured or unstructured format. | STORMS | | Yes | |

| | | | | | | |
|---|---|---|---|---|---|---|
| 1.1 | Study Design | State study design in abstract. | STORMS | See 3.0 for additional information on study design. | Yes | |
| 1.2 | Sequencing methods | State the strategy used for metagenomic classification. | STORMS | For example, targeted 16S by qPCR or sequencing, shotgun metagenomics, metatranscriptomics, etc. | Yes | |
| 1.3 | Specimens | Describe body site(s) studied. | STORMS | | Yes | |
| Introduction | | | | | | |
| 2.0 | Background and Rationale | Summarize the underlying background, scientific evidence, or theory driving the current hypothesis as well as the study objectives. | STORMS | | Yes | |
| 2.1 | Hypotheses | State the pre-specified hypothesis. If the study is exploratory, state any pre-specified study objectives. | STORMS | | Yes | Exploratory |
| Methods | | | | | | |
| 3.0 | Study Design | Describe the study design. | STORMS | Observational (Case-Control, Cohort, Cross-sectional survey, etc.) or Experimental (Randomized controlled trial, Non-randomized controlled trial, etc.). For a brief description of common study designs see: DOI: 10.11613/BM.2014.022<br>If applicable, describe any blinding (e.g. single or double-blinding) used in the course of the study. | Yes | |
| 3.1 | Participants | State what the population of interest is, and the method by which participants are sampled from that population. Include relevant information on physiological state of the subjects or stage in the life history of disease under study when participants were sampled. | STORMS | Examples of the population of interest could be: adults with no chronic health conditions, adults with type II diabetes, newborns, etc. This is the total population to whom the study is hoped to be generalizable to. The sampling method describes how potential participants were selected from that population.<br>If the participants are from a substudy of a larger study, provide a brief description of that study and cite that study.<br>Clearly state how cases and controls are defined.<br>An example of relevant physiological state might be pre/post menopausal for a vaginal microbiome study; examples of stage in the life history of disease could be whether specimens were collected during active or dormant disease, or before or after treatment. | Yes | Methods and Materials |

| | | | | | | |
|---|---|---|---|---|---|---|
| 3.2 | Geographic location | State the geographic region(s) where participants were sampled from. | MIxS: geographic location (country and/or sea,region) | Geographic coordinates can be reported to prevent potential ambiguities if necessary. | Yes | Methods and Materials |
| 3.3 | Relevant Dates | State the start and end dates for recruitment, follow-up, and data collection. | STORMS | Recruitment is the period in which participants are recruited for the study. In longitudinal studies, follow-up is the date range in which participants are asked to complete a specific assessment. Finally, data collection is the total period in which data is being collected from participants including during initial recruitment through all follow-ups. | Yes | Methods and Materials |
| 3.4 | Eligibility criteria | List any criteria for inclusion and exclusion of recruited participants. | Modified STROBE | Among potential recruited participants, how were some chosen and others not? This could include criteria such as sex, diet, age, health status, or BMI.<br>If there is a primary and validation sample, describe inclusion/exclusion criteria for each. | Yes | Methods and Materials |
| 3.5 | Antibiotics Usage | List what is known about antibiotics usage before or during sample collection. | STORMS | If participants were excluded due to current or recent antibiotics usage, state this here.<br>Other factors (e.g. proton pump inhibitors, probiotics, etc.) that may influence the microbiome should also be described as well. | Partly | Methods and Materials |
| 3.6 | Analytic sample size | Explain how the final analytic sample size was calculated, including the number of cases and controls if relevant, and reasons for dropout at each stage of the study. This should include the number of individuals in whom microbiome sequencing was attempted and the number in whom microbiome sequencing was successful. | STORMS | Consider use of a flow diagram (see template at https://stormsmicrobiome.org/figures). Also state sample size in abstract.<br>If power analysis was used to calculate sample size, describe those calculations. | Partly | Methods and Materials |
| 3.7 | Longitudinal Studies | For longitudinal studies, state how many follow-ups were conducted, describe sample size at follow-up by group or condition, and discuss any loss to follow-up. | STORMS | If there is loss to follow-up, discuss the likelihood that drop-out is associated with exposures, treatments, or outcomes of interest. | NA | |

| | | | | | | |
|---|---|---|---|---|---|---|
| 3.8 | Matching | For matched studies, give matching criteria. | Modified STROBE | "Matched" refers to matching between comparable study participants as cases and controls or exposed / unexposed.<br><br>Indicate whether participants were individual or frequency matched and in what ratio were they matched (e.g. 1 case to 1 control). | NA | |
| 3.9 | Ethics | State the name of the institutional review board that approved the study and protocols, protocol number and date of approval, and procedures for obtaining informed consent from participants. | STORMS | | Partly | Methods and Materials |
| 4.0 | Laboratory methods | State the laboratory/center where laboratory work was done. | STORMS | Provide a reference to complete lab protocols if previously published elsewhere such as on protocols.io. Note any modifications of lab protocols and the reason for protocol modifications. | Yes | Supplementary Methods 2 |
| 4.1 | Specimen collection | State the body site(s) sampled from and how specimens were collected. | MIxS: sample collection device or method; host body site | Use terms from the Uber-anatomy Ontology (https://www.ebi.ac.uk/ols/ontologies/uberon) to describe body sites in a standardized format. | Yes | Methods and Materials, Supplementary Methods 2 |
| 4.2 | Shipping | Describe how samples were stored and shipped to the laboratory. | STORMS | Include length of time from collection to receipt by the lab and if temperature control was used during shipping. | Yes | Supplementary Methods 2 |
| 4.3 | Storage | Describe how the laboratory stored samples, including time between collection and storage and any preservation buffers or refrigeration used. | STORMS | State where each procedure or lot of samples was done if not all in the same place.<br><br>Include reagent/lot/catalogue #s for storage buffers. | Yes | Baseclear, Supplementary Methods 2 |
| 4.4 | DNA extraction | Provide DNA extraction method, including kit and version if relevant. | MIxS: nucleic acid extraction | If any DNA quantification methods were used prior to DNA amplification or at the pooling step of library preparation, state so here. | Yes | Baseclear, Supplementary Methods 2 |
| 4.5 | Human DNA sequence depletion or microbial DNA enrichment | Describe whether human DNA sequence depletion or enrichment of microbial or viral DNA was performed. | STORMS | | NA | |
| 4.6 | Primer selection | Provide primer selection and DNA amplification methods as well as variable region sequenced (if applicable). | MIxS: pcr primers | | Yes | Supplementary Methods 2 |

| | | | | | | |
|---|---|---|---|---|---|---|
| 4.7 | Positive Controls | Describe any positive controls (mock communities) if used. | STORMS | If used, should be deposited under guidance provided in the 8.X items. | Yes | Supplementary Methods 2 |
| 4.8 | Negative Controls | Describe any negative controls if used. | STORMS | If used, should be deposited under guidance provided in the 8.X items. | Yes | Supplementary Methods 2 |
| 4.9 | Contaminant mitigation and identification | Provide any laboratory or computational methods used to control for or identify microbiome contamination from the environment, reagents, or laboratory. | STORMS | Includes filtering of reagents and other steps to minimize contamination. It is relevant to state whether the specimens of interest have low microbial load, which makes contamination especially relevant. | NA | Baseclear inclusion of negative water control and sterile lab environment |
| 4.10 | Replication | Describe any biological or technical replicates included in the sequencing, including which steps were replicated between them. | STORMS | Replication may be biological (redundant biological specimens) or technical (aliquots taken at different stages of analysis) and used in extraction, sequencing, preprocessing, and/or data analysis. | NA | |
| 4.11 | Sequencing strategy | Major divisions of strategy, such as shotgun or amplicon sequencing. | MIxS: sequencing method | For amplicon sequencing (for example, 16S variable region), state the region selected. State the model of sequencer used. | Yes | Methods and Materials |
| 4.12 | Sequencing methods | State whether experimental quantification was used (QMP/cell count based, spike-in based) or whether relative abundance methods were applied. | STORMS | These include read length, sequencing depth per sample (average and minimum), whether reads are paired, and other parameters. | NA | |
| 4.13 | Batch effects | Detail any blocking or randomization used in study design to avoid confounding of batches with exposures or outcomes. Discuss any likely sources of batch effects, if known. | STORMS | Sources of batch effects include sample collection, storage, library preparation, and sequencing and are commonly unavoidable in all but the smallest of studies. | NA | No randomization, batches are filled with samples according to the date they were received IMpACT and other studies, mixing control subjects and participants with ADHD |
| 4.14 | Metatranscriptomics | Detail whether any mRNA enrichment was performed and whether/how retrotranscription was performed prior to sequencing. Provide size range of isolated transcripts. Describe whether the sequencing library was stranded or not. Provide details on sequencing methods and platforms. | STORMS | Provide details on any internal standards which may have been used as well as parameters and versions of any software or databases used. | NA | |
| 4.15 | Metaproteomics | Detail which protease was used for digestion. Provide details on proteomic methods and platforms (e.g. LC-MS/MS, instrument type, column type, mass range, | STORMS | Provide details on any internal standards which may have been used as well as parameters and versions of any software or databases used. | NA | |

| | | | | | | |
|---|---|---|---|---|---|---|
| | | resolution, scan speed, maximum injection time, isolation window, normalised collision energy, and resolution). | | | | |
| 4.16 | Metabolomics | Specify the analytic method used (such as nuclear magnetic resonance spectroscopy or mass spectrometry). For mass spectrometry, detail which fractions were obtained (polar and/or non-polar) and how these were analyzed. Provide details on metabolomics methods and platforms (e.g. derivatization, instrument type, injection type, column type and instrument settings). | STORMS | Provide details on any internal standards which may have been used as well as parameters and versions of any software or databases used. | NA | |
| 5.0 | Data sources/ measurement | For each non-microbiome variable, including the health condition, intervention, or other variable of interest, state how it was defined, how it was measured or collected, and any transformations applied to the variable prior to analysis. | MIxS: host disease status | State any sources of potential bias in measurements, for example multiple interviewers or measurement instruments, and whether these potential biases were assessed or accounted for in study design.<br><br>Use terms from a standardized ontology such as the Experimental Factor Ontology (https://www.ebi.ac.uk/efo/) to describe variables of interest in a standardized format. | Yes | Methods and Materials, Supplementary Methods |
| 6.0 | Research design for causal inference | Discuss any potential for confounding by variables that may influence both the outcome and exposure of interest. State any variables controlled for and the rationale for controlling for them. | STORMS | For causal inference, this item refers to describing the assumptions that would be required to draw causal inferences from observational data. See Vujkovic-Cvijin, I., Sklar, J., Jiang, L. et al. Host variables confound gut microbiota studies of human disease. Nature 587, 448–454 (2020). https://doi.org/10.1038/s41586-020-2881-9 for more details on confounding in observational microbiome studies.<br><br>For example, hypothesized confounders may be controlled for by multivariable adjustment. Consider using a directed acyclic graph (DAG) to describe your causal model and justify any variables controlled for. DAGs can be made using www.dagitty.net. | NA | |
| 6.1 | Selection bias | Discuss potential for selection or survival bias. | STORMS | Selection bias can occur when some members of the target study population are more likely to be included in the study/final analytic sample than others. Some examples include survival bias (where part of the target study population is more likely to die before they can be studied), convenience sampling (where members of the target study population are not selected at random), and | NA | Feature selection was applied, while bias is introduced underestimating low prevalent features, the data-driven selection instead of hypothesis driven |

| | | | | | | |
|---|---|---|---|---|---|---|
| | | | | loss to follow-up (when probability of dropping out is related to one of the things being studied). | | might on the other hand reduce bias |
| 7.0 | Bioinformatic and Statistical Methods | Describe any transformations to quantitative variables used in analyses (e.g. use of percentages instead of counts, normalization, rarefaction, categorization). | STORMS | If a variable is analyzed using different transformations, state rationale for the transformation and for each analyses which version of the variable is used.<br><br>In case of any complex or multistep transformations, give enumerated instructions for reproducing those transformations. | Yes | Methods and Materials, Supplementary Methods 2 |
| 7.1 | Quality Control | Describe any methods to identify or filter low quality reads or samples. | MIxS: sequence quality check | If samples were excluded based on quality or read depth, list the criteria used, the number of samples excluded, and the final sample size after quality control. | Yes | Supplementary Methods 2 |
| 7.2 | Sequence analysis | Describe any taxonomic, functional profiling, or other sequence analysis performed. | MIxS: feature prediction; similarity search method | | Yes | Supplementary Methods 2 |
| 7.3 | Statistical methods | Describe all statistical methods. | Modified STROBE | Describe any statistical tests used, exploratory data analysis performed, dimension reduction methods/unsupervised analysis, alpha/beta metrics, and/or methods for adjusting for measurement bias.<br><br>If multiple statistical methods are possible, discuss why the methods used were selected.<br><br>If a multiple hypothesis testing correction method was used, describe the type of correction used.<br><br>State which taxonomic levels are analyzed. | Yes | Methods and Materials, Supplementary Methods |
| 7.4 | Longitudinal analysis | If the study is longitudinal, include a section that explicitly states what analysis methods were used (if any) to account for grouping of measurements by individual or patterns over time. | STORMS | | NA | |
| 7.5 | Subgroup analysis | Describe any methods used to examine subgroups and interactions. | STROBE | | NA | |

| | | | | | | |
|---|---|---|---|---|---|---|
| 7.6 | Missing data | Explain how missing data were addressed. | STROBE | "Missing data" refers to participant measurements such as covariates, exposures, outcomes, or time points that should have been collected but were not, not to zeros in taxonomic abundance tables or data points not applicable to that observation. | Yes | missing data was excluded |
| 7.7 | Sensitivity analyses | Describe any sensitivity analyses. | STROBE | | NA | |
| 7.8 | Findings | State criteria used to select findings for reporting. | STORMS | For example, false discovery rate with total number of tests, effect size threshold, significance threshold, microbes of interest. | Yes | Convergence across tools, FDR-corrected |
| 7.9 | Software | Cite all software (including read mapping software) and databases (including any used for taxonomic reference or annotating amplicons, if applicable) used. Include version numbers. | Modified STREGA | Installed packages, add-ons or libraries should be stated and cited in addition to the software used.<br>All parameters employed that differ from the default of that software/version should be provided.<br>This is in addition to, not a replacement for, publishing of code as outlined in the section Reproducible Research. | Yes | Methods and Materials, Supplementary Methods |
| 8.0 | Reproducible research | Make a statement about whether and how others can reproduce the reported analysis. | STORMS | Any protected information that has been excluded or provided under controlled access should be listed along with any relevant data access procedures. "On request from authors" is not sufficiently detailed; formal data access procedures and conditions should be defined.<br>If data are unavailable, state so clearly.<br>Consider using a specialized rubric for reproducible research (such as: https://mbio.asm.org/content/9/3/e00525-18.short).<br>Consider preregistering the study protocol (such as on osf.io or https://plos.org/open-science/preregistration/). | NA | Only open-source tools were applied and applications and settings deviating from default settings were described. Applied tools are cited in place. |
| 8.1 | Raw data access | State where raw data may be accessed including demultiplexing information. | STORMS | Robust, long-term databases such as those hosted by NCBI and EBI are preferred. If using a private repository, provide rationale. | NA | Raw sequences and demultiplexing information are stored on local servers, behavioral information is deposited in the dans (https://doi.org/10.17026/dans-xs2-nvp8). All data is available upon motivated request and must be dedicated to the research of ADHD. This is due to the |

| | | | | | | |
|---|---|---|---|---|---|---|
| | | | | | | informed consent form stating that collected data from the IMpACT2-NL study will only be used for the research of ADHD. |
| 8.2 | Processed data access | State where processed data may be accessed. | STORMS | Unfiltered data should be provided.<br>Robust, long-term databases such as those hosted by NCBI and EBI-EMBL are preferred. Repositories like zenodo (https://zenodo.org/) or publisso (https://www.publisso.de/en/working-for-you/doi-service/)<br>can be used to provide a DOI and long-term storage for processed datasets, even those which cannot be published openly. | NA | |
| 8.3 | Participant data access | State where individual participant data such as demographics and other covariates may be accessed, and how they can be matched to the microbiome data. | STORMS | If re-categorized, transformed, or otherwise derived variables were used in the analysis, these variables or code for deriving them should be provided.<br>Examples of how participant data can be matched to microbiome data are: using the same set of anonymized identifiers, or using different anonymized identifiers but providing a map.<br>Provided data should be sufficient to independently replicate the current analysis. | NA | |
| 8.4 | Source code access | State where code may be accessed. | STORMS | If a standard or formalized workflow was employed, reference it here. | Yes | Individual packages are all open access and cited in text |
| 8.5 | Full results | Provide full results of all analyses, in computer-readable format, in supplementary materials. | STORMS | For example, any fold-changes, p-values, or FDR values calculated, provided as a spreadsheet.<br>Use a machine-readable, plain-text format such as csv or tsv. | Yes | Results, Supplementary sesults |
| Results | | | | | | |
| 9.0 | Descriptive data | Give characteristics of study participants (e.g. dietary, demographic, clinical, social) and information on exposures and potential confounders. | STROBE | Typically reported in a table included in the paper or as a supplementary table. Indicate number of participants with missing data for each variable of interest.<br>This includes environmental and lifestyle factors that may affect the relationship between the microbiome and the condition of interest. Participant diet and medication use should be summarized, if known. | Yes | Results |

| | | | | | | |
|---|---|---|---|---|---|---|
| | | | | At minimum, age and sex of all participants should be summarized. | | |
| 10.0 | Microbiome data | Report descriptive findings for microbiome analyses with all applicable outcomes and covariates. | STORMS | This includes measures of diversity as well as relative abundances. These descriptive findings should be reported both for the sample overall and for individual groups. | Yes | Results, Supplementary Methods |
| 10.1 | Taxonomy | Identify taxonomy using standardized taxon classifications that are sufficient to uniquely identify taxa. | STORMS | If not using full taxonomic hierarchy, make sure it is clear whether names stated are species, genera, family, etc.<br><br>Italicize genus/species pairs. Consult journal guidelines or standardized references on taxonomic nomenclature. For instance, https://wwwnc.cdc.gov/eid/page/scientific-nomenclature | Yes | Genus only |
| 10.2 | Differential abundance | Report results of differential abundance analysis by the variable of interest and (if applicable) by time, clearly indicating the direction of change and total number of taxa tested. | STORMS | If there are more than two groups, include omnibus (multigroup) test results if applicable to the research question.<br><br>If applicable, reported effect sizes should include a measure of uncertainty such as the confidence interval. | Yes | Results, Supplementary Results |
| 10.3 | Other data types | Report other data analyzed--e.g. metabolic function, functional potential, MAG assembly, and RNAseq. | STORMS | | NA | |
| 10.4 | Other statistical analysis | Report any statistical data analysis not covered above. | STORMS | This could include subgroup analysis, sensitivity analyses, and cluster analysis.<br><br>Visualizations should be easily interpretable and colorblind-friendly. The caption and/or main text should provide a detailed description of visualizations for visually-impaired readers. | Yes | Results, Supplementary Results |
| Discussion | | | | | | |
| 11.0 | Key results | Summarise key results with reference to study objectives | STROBE | | Yes | Discussion |
| 12.0 | Interpretation | Give a cautious overall interpretation of results considering objectives, limitations, multiplicity of analyses, results from similar studies, and other relevant evidence. | STROBE | Define or clarify any subjective terms such as "dominant," "dysbiosis," and similar words used in interpretation of results.<br><br>When interpreting the findings, consider how the interpretation of the findings may be summarized or quoted for the general public such as in press releases or news articles.<br><br>If causal language is used in the interpretation (such as "alters," "affects," "results in," "causes," or "impacts"), assumptions made for causal inference | Yes | Discussion |

| | | | | | | |
|---|---|---|---|---|---|---|
| | | | | should be explicitly stated as part of 6.0 and 13.0.<br><br>Distinguish between function potential (ie inferred from metagenomics) and observed activity (ie metatranscriptomic, metabolomic, proteomic) if discussing microbial function. | | |
| 13.0 | Limitations | Discuss limitations of the study, taking into account sources of potential bias or imprecision. | STROBE | Also consider limitations resulting from the methods (especially novel methods), the study design, and the sample size. | Yes | Discussion |
| 13.1 | Bias | Discuss any potential for bias to influence study findings. | STORMS | May include sampling method, representativeness of study participants, or potential confounding. | Yes | Discussion |
| 13.2 | Generalizability | Discuss the generalisability (external validity) of the study results | STROBE | To what populations or other settings do you expect the conclusions to generalize? | NA | |
| 14.0 | Ongoing/future work | Describe potential future research or ongoing research based on the study's findings. | STORMS | | Yes | Discussion |
| Other information | | | | | | |
| 15.0 | Funding | Give the source of funding and the role of the funders for the present study and, if applicable, for the original study on which the present article is based | STROBE | | Yes | Acknowledgements |
| 15.1 | Acknowledgements | Include acknowledgements of those who contributed to the research but did not meet critera for authorship. | STORMS | For general guidelines on authorship, see http://www.icmje.org and https://www.elsevier.com/authors/journal-authors/policies-and-ethics/credit-author-statement | NA | |
| 15.2 | Conflicts of Interest | Include a conflicts of interest statement. | STORMS | | Yes | Conflict of Interest/Disclosure |
| 16.0 | Supplements | Indicate where supplements may be accessed and what materials they contain. | STORMS | | Yes | In text, where applicable |
| 17.0 | Supplementary data | Provide supplementary data files of results with for all taxa and all outcome variables analyzed. Indicate the taxonomic level of all taxa. | STORMS | Depending on the analysis performed, examples of the supplemental results included could be mean relative abundance, differential abundance, raw p-value, multiple hypothesis testing-adjusted p-values, and standard error.<br><br>All discussed taxa should include the taxonomic level (e.g. class, order, genus). | Yes | Supplementary Methods & results in supplementary material file |

## References

(1) Raine, A.; Dodge, K.; Loeber, R.; Gatzke-Kopp, L.; Lynam, D.; Reynolds, C.; Stouthamer-Loeber, M.; Liu, J. The reactive–proactive aggression questionnaire: Differential correlates of reactive and proactive aggression in adolescent boys. *Aggressive Behavior: Official Journal of the International Society for Research on Aggression* **2006**, *32* (2), 159-171.

(2) Bloemendaal, M.; Vlaming, P.; de Boer, A.; Vermeulen-Kalk, K.; Bouman, A.; Kleefstra, T.; Arias Vasquez, A. The role of the gut microbiota in patients with Kleefstra syndrome. *American Journal of Medical Genetics Part B: Neuropsychiatric Genetics* **2023**.

(3) Callahan, B. J.; McMurdie, P. J.; Rosen, M. J.; Han, A. W.; Johnson, A. J. A.; Holmes, S. P. DADA2: High-resolution sample inference from Illumina amplicon data. *Nature methods* **2016**, *13* (7), 581-583.

(4) Gloor, G. B.; Macklaim, J. M.; Pawlowsky-Glahn, V.; Egozcue, J. J. Microbiome datasets are compositional: and this is not optional. *Frontiers in microbiology* **2017**, *8*, 2224.

(5) Nearing, J. T.; Douglas, G. M.; Hayes, M. G.; MacDonald, J.; Desai, D. K.; Allward, N.; Jones, C. M.; Wright, R. J.; Dhanani, A. S.; Comeau, A. M. Microbiome differential abundance methods produce different results across 38 datasets. *Nature Communications* **2022**, *13* (1), 342.

(6) Lahti, L.; Shetty, S. microbiome R package. **2017**.

(7) Kloke, J. D.; McKean, J. W. Rfit: rank-based estimation for linear models. *R J.* **2012**, *4* (2), 57.

(8) Oksanen, J.; Kindt, R.; Legendre, P.; O'Hara, B.; Stevens, M. H. H.; Oksanen, M. J.; Suggests, M. The vegan package. *Community ecology package* **2007**, *10* (631-637), 719.

(9) Meinshausen, N.; Bühlmann, P. Stability selection. *Journal of the Royal Statistical Society: Series B (Statistical Methodology)* **2010**, *72* (4), 417-473.
